# A Mathematical Model Supporting a Hyperpredation Effect in the Apparent Competition Between Invasive Eastern Cottontail and Native European Hare

**DOI:** 10.1007/s11538-021-00873-9

**Published:** 2021-03-27

**Authors:** Elisa Caudera, Simona Viale, Sandro Bertolino, Jacopo Cerri, Ezio Venturino

**Affiliations:** 1 Dipartimento di Matematica “Giuseppe Peano”, Università di Torino, via Carlo Alberto 10, 10123 Torino, Italy; 2 Dipartimento di Scienze della Vita e Biologia dei Sistemi, Università di Torino, Via Accademia Albertina 13, 10123 Torino, Italy

**Keywords:** Predator–prey, Competition, Predation, Invasion, Equilibria, Bifurcation, 92D25, 92D40

## Abstract

In this work a mathematical model is built in order to validate on theoretical grounds field study results on a three-species system made of two prey, of which one is native and another one invasive, together with a native predator. Specifically, our results mathematically describe the negative effect on the native European hare after the introduction of the invasive Eastern cottontail, mediated by an increased predation rate by foxes. Two nonexclusive assumptions can be made: an increase in cottontail abundance would lead to a larger fox population, magnifying their predatory impact (“hyperpredation”) on hares; alternatively, cottontails attract foxes in patches where they live, which are also important resting sites for hares and consequently the increased presence of foxes results in a higher predation rates on hares. The model results support hyperpredation of increasing fox populations on native hares.

## Introduction

Biological invasions—i.e. the human-mediated introduction of species outside their native range—represent one of the main drives of global change. Introduced species change ecosystems composition and species interaction, threatening native biodiversity and representing a major source of extinction (Clavero and García-Berthou 2005; Kumschick et al. 2015). Introduced species may interact negatively with native ones, through numerous, such as competition, predation, hybridization, the transmission of disease, see IUCN 2020 for a complete list of impact mechanisms (IUCN EICAT Categories and Criteria 2020). Competition is the interaction between species of the same trophic level, which leads to negative consequences for one of the two. It can be regarded as the direct or indirect interaction of organism that leads to a change in fitness when they share the same resources, such as food or nesting sites (Holomuzki et al. 2010). The reduction in the fitness of individuals translates at the population level into a change in demography which could determine the decline or the extinction of one of the two species. There are three major mechanisms of competition. Interference competition and exploitation competition are categorized as real competition. Interference competition involves direct interactions between individuals. For example, in Ferretti et al. (2011) it is reported the negative effects of behavioural interference by fallow deer (*Dama dama*) on the foraging behaviour of roe deer (*Capreolus capreolus*). Exploitation competition implies indirect negative interactions between two species arising from the use of a common resource (Schoener 1983). A classic example is the competition between the introduced Eastern grey squirrel (*Sciurus carolinesis*) and the native Eurasian red squirrel (*Sciurus vulgaris*). The replacement is mainly due to exploitation competition for food resources, with the introduced species more efficient in their use (Wauters et al. 2002a, b). The third form of competition is more complex and concerns species that do not interact directly and do not exploit the same resources, but still influence each other through shared enemies, such as predators, parasites, or pathogens (Holt and Bonsall 2017). In this case, the competition is mediated by the action of a third species of another trophic level. This species might be a pathogen: in Great Britain, grey squirrels carry a squirrel pox virus which is lethal for the red squirrel (Tompkins et al. 2003; Romeo et al. 2019). The virus carried by the introduced species has a detrimental effect on the native one, increasing the replacement speed between the two species. However, the common enemy might also be a predator: in New Zealand, introduced rabbits (*Oryctolagus cuniculus*) created large populations of mammal predators, which also prey upon native lizards (*Oligosoma* spp. (Norbury 2001).

The Eastern cottontail (*Sylvilagus floridanus*) is a lagomorph native to the American continent that was introduced in Italy in the 1960s for hunting purposes (Bertolino et al. 2011a). The species is now widespread in Central and Northern Italy (Bertolino et al. 2011a; Loy et al. 2019) where it competes with the native European hare (*Lepus europaeus*). The two species select different macro- and microhabitats, both for resting and during feeding activity (Bertolino et al. 2011a, 2013). Cottontails carry several viruses and parasites, which can potentially affect hares (Bertolino et al. 2010; Tizzani et al. 2014); however, competitive interactions mediated by parasites have not yet been highlighted. Recently, in Cerri et al. (2017) an apparent competition between Eastern cottontail and European hare mediated by the fox (*Vulpes vulpes*) as a common predator is shown. Introduced cottontails affect prey–predator dynamics of native hares and foxes: the abundance of foxes is positively associated with the one of hares, when cottontails are scarce, suggesting the main influence of external factors, such as habitat quality. However, this association becomes increasingly negative as cottontails increase in their abundance. When cottontails are abundant, increases in the abundance of foxes are negatively correlated with the abundance of European hares (see Fig. 2 in Cerri et al. (2017)). This pattern is suggestive of an indirect competition between cottontails and hares mediated by fox predation.

Starting from the work of Cerri et al. (2017), we introduce here a mathematical model to simulate this three-species system and investigate it, to possibly validate on theoretical grounds the results of the field study (Cerri et al. 2017).

The paper is organized as follows. After the model formulation in the next section, its equilibria are assessed in Sect. 3. The three-species system behaviour is analysed in detail in Sect. 4, numerical simulations are reported in Sect. 5, and a biological discussion sets these results in the ecological perspective, in Sect. 6.

## Model Formulation

The dynamical system that we propose can be described by the following equations, where all the parameters are nonnegative and where the parameter *r* generally interpreted as the traditional growth rate of the logistic equation, represents instead the foxes reproduction. This important difference should be remarked throughout the paper.

1 $$\begin{aligned} \begin{aligned}&{\dot{V}}=V \left( r-c_{VV} V-m+eaS+ebL \right) \\&{\dot{S}}=S \left( s-c_{SS} S-n-aV \right) \\&{\dot{L}}=L \left( u-c_{LL} L-p-bV \right) \end{aligned} \end{aligned}$$

The reason for separating the reproduction *r* and the mortality *m* rates for the foxes, and more in general for the various model populations, and not using the standard logistic equation, lies in the fact that we are going to use field data, for which, in principle, the condition of the positivity of the net reproduction rate may not be satisfied, and furthermore, that we would like to contemplate the possibility of disappearing populations.

We also use the Holling type (HT) I response function for hunting because the density of the foxes is very low, while hares and cottontails do not attain very large numbers. Indeed, although not much is known about the foxes density in Italy, a reasonable range per square kilometre is usually taken as [1.0, 2.5], (Boitani and Prigioni 2003). The maximal observed cottontail density is 110 per km$^2$ (Bertolino et al. 2011a), while for hares it ranges in the interval [26, 40], (Pandini et al. 1998). Therefore, a feeding saturation phenomenon for foxes does not arise, and a simple bilinear term is sufficient to describe the interactions, as it represents a linear approximation in the meaningful range of the HTII response function describing satiation.

The first equation in (1) describes the dynamics of the red fox *Vulpes vulpes* *V* that grows logistically with reproduction rate *r*, death rate *m*, intraspecific competition rate $c_{VV}$ and gets a benefit from the capture of its prey scaled by a conversion coefficient *e*, related to the digestibility of the prey, which, for foxes, lies in the range [0.8958, 0.9190], when predating on rabbits (Maurya et al. 2012), while hunting hares, it is 0.910 (Ruehe et al. 2008). As these figures are similar, we take the conversion factor *e* to be the same for the two kinds of prey.

The second equation describes the dynamics of the Eastern cottontail *Silvilagus floridanus*, *S*, that also grows logistically with reproduction rate *s*, mortality rate *n*, has the intraspecific competition rate $c_{SS}$ and is hunted by the foxes at rate *a*.

The last equation describes the dynamics of the European hare *Lepus europaeus*, *L*. The logistic growth occurs with reproduction rate *u*, mortality rate *p*, intraspecific competition rate $c_{LL}$. Hares are captured by foxes at rate *b*. In Table 1 we summarize the meaning of each model parameter.

Note that no direct competition between the invasive cottontails and the native hares is here considered, as on biological grounds the two species occupy different ecological niches and thus do not directly interfere with each other (Bertolino et al. 2011a, b, 2013). Rather, it is hypothesized that their interaction occurs through indirect competition mediated by the red fox.

The model (1) contains the predators’ interference on the two types of prey, but the latter occupy different ecological niches (Bertolino et al. 2011a, b, 2013), for which, in the absence of the foxes, each one individually or both together settle to their respective carrying capacities, that are explicitly given by the nonvanishing population values contained in the equilibria $E_1$, $E_2$, $E_4$ and $E_6$ below. When foxes are present and one prey is absent, the population levels attained are clearly modified by their mutual interactions, see equilibria $E_3$, $E_5$.

For the later study of the equilibria stability, we will need the Jacobian of the system (1):

$$\begin{aligned} J= \begin{bmatrix} r-2 c_{VV} V -m+eaS+ebL &{} eaV &{} ebV\\ -aS &{} s-2 c_{SS} S -n-aV &{} 0\\ -bL &{} 0 &{} u-2 c_{LL} L -p-bV \end{bmatrix} . \end{aligned}$$

**Table 1:** Model parameters

*r*	Foxes reproduction rate
*s*	Cottontails reproduction rate
*u*	Hares reproduction rate
*m*	Foxes mortality rate
*n*	Cottontails mortality rate
*p*	Hares mortality rate
$c_{VV}$	Foxes intraspecific competition rate
$c_{SS}$	Cottontails intraspecific competition rate
$c_{LL}$	Hares intraspecific competition rate
*e*	Captured prey conversion coefficient
*a*	Foxes hunting rate on cottontails
*b*	Foxes hunting rate on hares

## Model Analysis

### Boundedness

In this subsection we show that the system trajectories are confined in a compact set in the first quadrant of the phase space. This result is relevant from the ecological point of view, because it says that in the presence of finite resources, no population can grow without limit.

The result is obtained by considering all the living individuals in the model. Let us define the total environmental population $A=V+S+L$. Because each population is nonnegative, if *A* is bounded, then also *V*, *S* and *L* are bounded. The details for which boundedness of *A* holds are reported in “Appendix”.

### Equilibria

System (1) has the following eight possible equilibria $E_i=(V_i,S_i,L_i)$, for $i=0,\ldots ,7$. The origin, $E_0 = (0,0,0)$, corresponds to extinction of the whole three-species system. The three points in which only one population thrives:

$$\begin{aligned} E_1 = \left( \frac{r-m}{c_{VV}},0,0\right) , \quad E_2 = \left( 0,\frac{s-n}{c_{SS}},0\right) , \quad E_4 = \left( 0,0,\frac{u-p}{c_{LL}}\right) . \end{aligned}$$

The equilibria with only one vanishing population:

$$\begin{aligned} E_3= & {} \left( \frac{ae(s-n)+c_{SS}(r-m)}{a^2e+c_{VV}c_{SS}},\frac{a(m-r)+c_{VV}(s-n)}{a^2e+c_{VV}c_{SS}},0\right) , \\ E_5= & {} \left( \frac{be(u-p)+c_{LL}(r-m)}{b^2e+c_{VV}c_{LL}},0,\frac{b(m-r)+c_{VV}(u-p)}{b^2e+c_{VV}c_{LL}}\right) , \\ E_6= & {} \left( 0,\frac{s-n}{c_{SS}},\frac{u-p}{c_{LL}}\right) \end{aligned}$$

and coexistence $E_7 = (V_7, S_7, L_7)$ whose population levels can explicitly be evaluated:

$$\begin{aligned} V_7= & {} \frac{bec_{SS}(u-p)+aec_{LL}(s-n)+c_{SS}c_{LL}(r-m)}{b^2ec_{SS}+a^2ec_{LL}+c_{VV}c_{SS}c_{LL}}\\ S_7= & {} \frac{abe(p-u)+b^2e(s-n)+ac_{LL}(m-r)+c_{VV}c_{LL}(s-n)}{b^2ec_{SS}+a^2ec_{LL}+c_{VV}c_{SS}c_{LL}}\\ L_7= & {} \frac{a^2e(u-p)+abe(n-s)+bc_{SS}(m-r)+c_{VV}c_{SS}(u-p)}{b^2ec_{SS}+a^2ec_{LL}+c_{VV}c_{SS}c_{LL}} \end{aligned}$$

While the origin is always feasible, it turns out to be conditionally stable, in view of the fact that the Jacobian reduces to a diagonal matrix from which the eigenvalues are immediate and provide the following stability conditions:

2 $$\begin{aligned} r<m, \quad s<n, \quad u<p. \end{aligned}$$

Note that these conditions imply indeed that mortalities exceed the reproduction rates, entailing the extinction of each and every species.

For $E_i$, $i=1,2,4$, the feasibility conditions are, respectively,

3 $$\begin{aligned} r\ge m; \quad s\ge n; \quad u \ge p. \end{aligned}$$

For $E_1$ stability hinges on the following inequalities, $m<r$, which is implied by feasibility and is therefore redundant, and:

4 $$\begin{aligned} s<n+aV_1, \quad u<p+bV_1. \end{aligned}$$

For $E_2$ we find instead the stability conditions

5 $$\begin{aligned} r+eaS_2<m, \quad u<p, \end{aligned}$$

and $n<s$, which follows from feasibility.

For $E_4$, which is feasible if the hares population is nonnegative, i.e. the last condition in (3) that ensures also the negativity of the last eigenvalue, the stability conditions come from the other two explicit eigenvalues and give:

6 $$\begin{aligned} r+ebL_4<m, \quad s<n. \end{aligned}$$

For the equilibria with two nonvanishing populations, the results are as follows.

$E_3$ is feasible for

7 $$\begin{aligned} aes+c_{SS}r \ge aen+c_{SS}m, \quad am+c_{VV}s \ge ar+c_{VV}n. \end{aligned}$$

Here one eigenvalue factorizes, namely $J_{33}(E_3)=u-p-V_3$. The remaining submatrix has negative trace and positive determinant; hence, the Routh–Hurwitz conditions are satisfied and thus, its eigenvalues are negative. Stability reduces to requiring

8 $$\begin{aligned} u<p+bV_3. \end{aligned}$$

At $E_5$ two populations need to be nonnegative, giving for feasibility

9 $$\begin{aligned} beu+c_{LL}r \ge bep+c_{LL}m, \quad bm+c_{VV}u \ge br+c_{VV}p. \end{aligned}$$

As for $E_3$, here one eigenvalue can be factorized, $J_{22}(E_5)=s-n-aV_5$. The remaining minor has negative trace and positive determinant, and therefore, its eigenvalues are negative. The only stability condition is:

10 $$\begin{aligned} s<n+aV_5. \end{aligned}$$

For $E_6$ feasibility follows by satisfying

11 $$\begin{aligned} s \ge n, \quad u \ge p \end{aligned}$$

and all the Jacobian eigenvalues are explicitly known, $r-m+eaS_6+ebL_6$, $-s c_{SS} <0$, $-u c_{LL} <0$. Thus, just the first one ensures stability, namely:

12 $$\begin{aligned} r+eaS_6+ebL_6<m. \end{aligned}$$

Feasibility for $E_7$ entails the following inequalities:

13 $$\begin{aligned}&bec_{SS}u+aec_{LL}s+c_{SS}c_{LL}r \ge bec_{SS}p+aec_{LL}n+c_{SS}c_{LL}m abep \nonumber \\&\quad +b^2es+ac_{LL}m+c_{VV}c_{LL}s \ge abeu+b^2en+ac_{LL}r+c_{VV}c_{LL}n a^2eu\nonumber \\&\quad +aben+bc_{SS}m+c_{VV}c_{SS}u \ge a^2ep+abes+bc_{SS}r+c_{VV}c_{SS}p \end{aligned}$$

This equilibrium, when feasible, turns out to be unconditionally stable. Indeed, using the Routh–Hurwitz criterion, for the trace we find:

$$\begin{aligned} -{\text {tr}}(J(E_7))=c_{VV} V_7 +c_{SS} S_7 +c_{LL} L_7 >0 \end{aligned}$$

and for the determinant:

$$\begin{aligned} -\det (J(E_7)) = \left( c_{VV}c_{SS}c_{LL}+b^2e c_{SS}+ea^2 c_{LL}\right) V_7S_7L_7>0. \end{aligned}$$

Further, we need to assess the sum $M_2^{(E_7)}$ of the principal minors of order 2:

$$\begin{aligned}&M_2^{(E_7)}=c_{VV}c_{SS} V_7 S_7 + c_{VV}c_{LL} V_7 L_7 \\&\quad + c_{SS}c_{LL} S_7 L_7 +eb^2L_7V_7+ea^2S_7V_7. \end{aligned}$$

The final Routh–Hurwitz condition for stability is then:

14 $$\begin{aligned} -{\text {tr}}(J(E_7)) M_2 > -\det (J(E_7)) . \end{aligned}$$

This condition is always satisfied, as it reduces to

$$\begin{aligned}&(c_{VV} V_7 +c_{SS} S_7 )(c_{VV}c_{SS}+ea^2) V_7 S_7 +eb^2L_7V_7 (c_{VV} V_7 +c_{LL} L_7 )\\&\quad + (c_{VV} V_7 +c_{SS} S_7 +c_{LL} L_7 ) (c_{VV}c_{LL} V_7 L_7 + c_{SS}c_{LL} S_7 L_7 ) > 0 \end{aligned}$$

**Table 2:** Equilibria feasibility and stability

Equilibrium	Feasibility	Stability
$E_0$	−	$r<m$, $s<n$, $u<p$
$E_1$	$m \le r$	$m<r$, $s<n+aV_1$, $u<p+bV_1$
$E_2$	$n \le s$	$r+eaS_2<m$, $n<s$, $u<p$
$E_3$	$aes+c_{SS}r \ge aen+c_{SS}m$,	$u<p+bV_3$
	$$am+c_{VV}s \ge ar+c_{VV}n$$	
$E_4$	$p \le u$	$r+ebL_4<m$, $s<n$, $p<u$
$E_5$	$beu+c_{LL}r \ge bep+c_{LL}m$,	$s<n+aV_5$
	$$bm+c_{VV}u \ge br+c_{VV}p$$	
$E_6$	$n \le s$, $p \le u$	$r+eaS_6+ebL_6<m$
$E_7$	(13)	Stable

## The Interactions Behaviour

### Equilibria Global Stability

The conditions summarized in Table 2 suggest that there are pairs of equilibria that cannot occur simultaneously. This will be better investigated in the next subsection, but since bi- or multistability among some subsets of equilibria is prevented by these conditions, it is worth to investigate their possible global stability. To this end, we construct suitable Lyapunov function candidates ${\mathcal {L}}_i (V,S,L)$ for each equilibrium, such that

$$\begin{aligned} \frac{{\text {d}}{\mathcal {L}}_i}{{\text {d}}t}=P^T A_i P+\alpha _i(V,S,L), \end{aligned}$$

where $\alpha _i(V,S,L)<0$ for equilibrium $E_i$, $P=(V-V_i,S-S_i,L-L_i)^T$ and *A* is negative definite, so that finally the Lyapunov conditions: ${\mathcal {L}}(V,S,L) \ge 0$, $(V,S,L)\in {{\mathbf {R}}_+^3}$, i.e. nonnegativity, in the first orthant, as well as

$$\begin{aligned} {\mathcal {L}}_i(E_i)=0, \quad \frac{{\text {d}}{\mathcal {L}}_i}{{\text {d}}t} <0 \end{aligned}$$

are satisfied.

It turns out that all equilibria are globally asymptotically stable, whenever they are locally asymptotically stable. In fact it turns out that the matrix $A_i$ is independent of the equilibrium that is considered, $i=1,\ldots ,7$; it has namely the following structure:

15 $$\begin{aligned} A_i= A= \begin{bmatrix} -c c_{VV} &{} \quad a\frac{ce-g}{2} &{}\quad b\frac{ce-f}{2}\\ a\frac{ce-g}{2} &{} \quad -s c_{SS} &{}\quad 0\\ b\frac{ce-f}{2} &{} \quad 0 &{} \quad -f c_{LL} \end{bmatrix} . \end{aligned}$$

The mathematical details are deferred to “Appendix”.

### Bifurcations

In view of the results of the previous subsection, Hopf bifurcations are forbidden, a fact that prevents the onset of persistent oscillations in the model solution trajectories. Indeed, at every equilibrium, either the eigenvalues are all explicitly known and real, or, when the Routh–Hurwitz conditions are used, in case of equilibria $E_3$ and $E_5$, the trace can never vanish, and this prevents the possibility of having pure imaginary eigenvalues. In case of $E_7$ instead, it is the condition (14) that cannot vanish. In saying so, we of course exclude, as biologically unrealistic, the very particular cases in which some or all the reproduction rates of the three species vanish. Therefore, only transcritical bifurcations could then relate the various system’s equilibria to each other. We will now rigorously explore this issue. The mathematical tool is represented by Sotomayor’s theorem, (Perko 2001).

Denoting by $F=(F_1,F_2,F_3)^T$ the right-hand side of (1), with $F_i=F_i(V,S,L)$, we first evaluate its partial derivatives with respect to the model parameters:

$$\begin{aligned} F_a= & {} \left( eSV,-SV,0 \right) ^T, \quad F_b= \left( eLV,0,-LV \right) ^T, \quad F_r= \left( V,0,0 \right) ^T, \quad F_s= \left( 0,S,0 \right) ^T, \\ F_u= & {} \left( 0,0,L \right) ^T, \quad F_{c_{VV}}= \left( -V^2,0,0 \right) ^T, \quad F_{c_{SS}}= \left( 0,-S^2,0 \right) ^T, \quad F_{c_{LL}}= \left( 0,0,-L^2 \right) ^T . \end{aligned}$$

We then need the Jacobians of the above vector-valued functions:

$$\begin{aligned} DF_a= & {} \begin{bmatrix} eS &{} eV &{} 0\\ -S &{} -V &{} 0\\ 0 &{} 0 &{} 0 \end{bmatrix}, \quad DF_b= \begin{bmatrix} eL &{} 0 &{} eV\\ 0 &{} 0 &{} 0\\ -L &{} 0 &{} -V \end{bmatrix}, \quad DF_r= \begin{bmatrix} 1 &{} 0 &{} 0\\ 0 &{} 0 &{} 0\\ 0 &{} 0 &{} 0 \end{bmatrix}, \quad DF_s= \begin{bmatrix} 0 &{} 0 &{} 0\\ 0 &{} 1 &{} 0\\ 0 &{} 0 &{} 0 \end{bmatrix}, \\ DF_u= & {} \begin{bmatrix} 0 &{} 0 &{} 0\\ 0 &{} 0 &{} 0\\ 0 &{} 0 &{} 1 \end{bmatrix}, \quad DF_{c_{VV}}= \begin{bmatrix} -2V &{} 0 &{} 0\\ 0 &{} 0 &{} 0\\ 0 &{} 0 &{} 0 \end{bmatrix}, \quad DF_{c_{SS}}= \begin{bmatrix} 0 &{} 0 &{} 0\\ 0 &{} -2S &{} 0\\ 0 &{} 0 &{} 0 \end{bmatrix}, \quad DF_{c_{LL}}= \begin{bmatrix} 0 &{} 0 &{} 0\\ 0 &{} 0 &{} 0\\ 0 &{} 0 &{} -2L \end{bmatrix} . \end{aligned}$$

Then, in order to evaluate $D^2 F$ we need:

$$\begin{aligned} F^1_{VV}= & {} -2c_{VV}, \quad F^1_{VS}=ea, \quad F^1_{VL}=eb, \quad F^1_{SS}=0, \quad F^1_{SL}=0, \quad F^1_{LL}=0, \\ F^2_{VV}= & {} 0, \quad F^2_{VS}=-a, \quad F^2_{VL}=0, \quad F^2_{SS}=-2c_{SS}, \quad F^2_{SL}=0, \quad F^2_{LL}=0, \\ F^3_{VV}= & {} 0, \quad F^3_{VS}=0, \quad F^3_{VL}=-b, \quad F^3_{SS}=0, \quad F^3_{SL}=0, \quad F^3_{LL}=-2c_{LL} . \end{aligned}$$

Further, to evaluate $D^3 F$ we need the partial derivatives of order three, but it can easily be assessed that they all vanish. As a consequence, system (1) is therefore not satisfying the necessary condition for a pitchfork bifurcation:

$$\begin{aligned} \mathbf{w }^T\left[ D^3 F (\mathbf{x }_0,\mu _0)(\mathbf{v },\mathbf{v },\mathbf{v })\right] \ne 0. \end{aligned}$$

Therefore, all pitchfork bifurcations are ruled out and so possibly only saddle-node or transcritical bifurcations can arise.

**Fig. 1:**
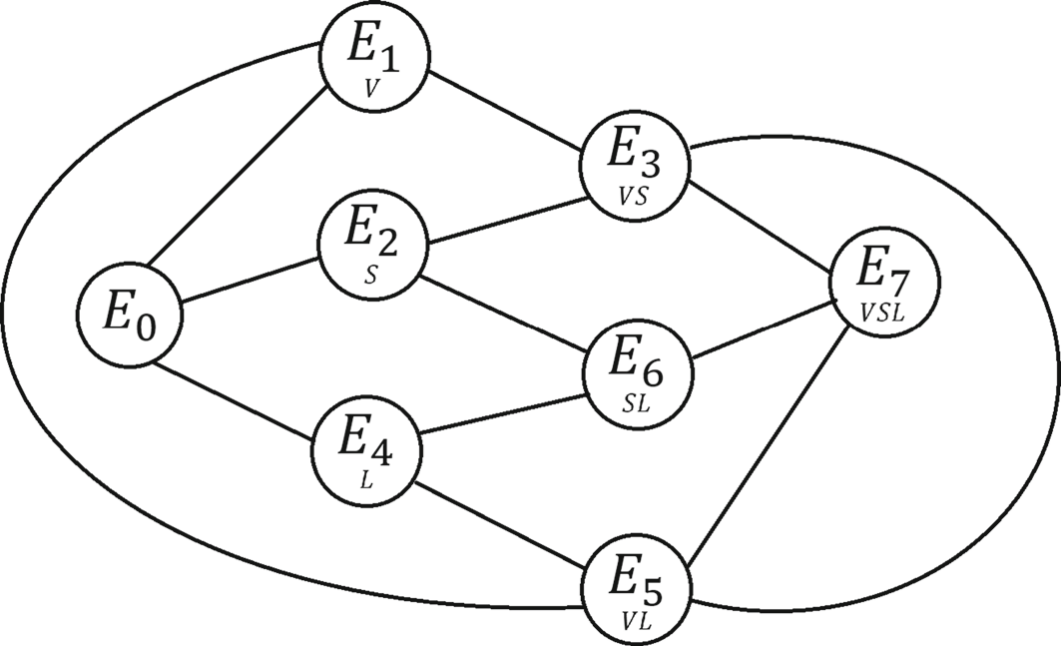
(Color Figure Online) The equilibria of the system (1). The nodes in the graph denoted the equilibria in which the letter subscripts denote the nonvanishing populations; the arcs denote the possible transcritical bifurcations that connect them

The mathematical details for this analysis are reported in the “Appendix”, and as we will see, only the latter are indeed found. A picture of the structure of all the analytically found bifurcations is reported in Fig. 1.

### Biological Interpretation

We now provide some insights on the situation, based on all the previous findings, in terms of some relevant biological quantities. In particular, we will focus on the net reproduction rates of each species

$$\begin{aligned} r-m, \quad s-n, \quad u-p, \end{aligned}$$

and some additional quantities, namely:

$$\begin{aligned} k_1:= a\frac{r-m}{s-n}, \quad k_2:= b\frac{r-m}{u-p}, \quad h_2:=ae\frac{s-n}{m-r}, \quad g_2:=be\frac{u-p}{m-r}. \end{aligned}$$

In addition, recall that bistability is forbidden. Thus, in the following discussion, as always the equilibria with some of the vanishing populations appear, coexistence is impossible for all the listed situations.

- When all the net reproduction rates are negative
  $$\begin{aligned} r<m, \quad s<n, \quad u<p \end{aligned}$$
  only $E_0$ is feasible and stable; therefore, as intuition suggests, all the species are driven to extinction.
- For the foxes reproducing effectively only $E_1$ is feasible and stable:
  $$\begin{aligned} m<r, \quad s<n, \quad u<p. \end{aligned}$$
  This makes sense, as foxes are supposed to have other feeding resources.
- When
  $$\begin{aligned} r<m, \quad n<s, \quad u<p \end{aligned}$$
  $E_2$ is feasible, but stable only if $c_{SS}<h_2$, while in the opposite case $c_{SS}>h_2$, $E_3$ is feasible and stable. In this situation, the cottontails thrive alone if their intraspecific competition is low enough, quite intuitively; otherwise, they would support also the foxes and the two species survive together.
- Instead, if
  $$\begin{aligned} m<r, \quad n<s, \quad u<p \end{aligned}$$
  we find only the equilibria $E_1$ and $E_3$. The former is feasible and stable only if $c_{VV}<k_1$, the latter for feasibility needs $c_{VV}>k_1$. Thus, here either the foxes survive alone, if their intraspecific competition is low enough; otherwise, they coexist with the cottontails.
- For
  $$\begin{aligned} r<m, \quad s<n, \quad p<u \end{aligned}$$
  the possible equilibria are $E_4$ and $E_5$. $E_4$ is unconditionally feasible, but it is stable only if $c_{LL}<g_2$. Conversely, for $c_{LL}>g_2$, $E_5$ becomes feasible and in such case it is stable. Thus, here either the hares survive alone, provided their intraspecific competition falls below a threshold; otherwise, they thrive together with the generalist predators, the foxes.
- When
  $$\begin{aligned} m<r, \quad s<n, \quad p<u \end{aligned}$$
  $E_1$ is feasible but stable only if $c_{VV}<k_2$, while for the opposite condition, $E_5$ is feasible and always stable. Here for a small enough intraspecific competition, the foxes thrive alone, wiping out both hares and cottontails, but in the opposite case the hares also survive.
- Whenever
  $$\begin{aligned} r<m, \quad n<s, \quad p<u \end{aligned}$$
  foxes and cottontails coexist at $E_3$, or the foxes thrive with the hares at $E_5$ or else foxes disappear and only cottontails and hares share the environment. More precisely, $E_3$ is feasible if $c_{SS}<h_2$ and stable if $u<p+bV_3$, $E_5$ is feasible if $c_{LL}<g_2$ and stable for $s<n+aV_5$, $E_6$ is unconditionally feasible but stable only if $r+eaS_6+ebL_6<m$.
- Finally, for
  $$\begin{aligned} m<r, \quad n<s, \quad p<u \end{aligned}$$
  only one population survives unconditionally in the environment, the foxes; here all possible alternative equilibria are feasible, and only their stability determines the three-species interaction outcome. More precisely, the foxes wipe out the other two populations, equilibrium $E_1$, if $c_{VV}<k_1$ and $c_{VV}<k_2$, they thrive with the cottontails at $E_3$ if $c_{SS}>h_2$, $c_{VV}<k_1$ and $u<p+bV_3$, these conditions, respectively, arising for feasibility and stability, or finally, they survive together with the hares, equilibrium $E_5$ which is feasible for $c_{VV}<k_2$ and $c_{LL}<g_2$, while stable in case $s<n+aV_5$.

To better illustrate how the species at steady state are related to each other, we provide Fig. 1, where the graph contains as nodes the equilibria, whose nonvanishing populations are denoted by subscripts, and the arcs their connecting transcritical bifurcations.

## Numerical Simulations

Although the demography of three-species system has been completely characterized analytically, it is worth to see also some simulations with realistic parameter values, in order to compare their results with the field findings of the biologists.

### Parameter Values

Some of the parameter values are taken from as well as from (Amori et al. 2008; Barbara et al. 2018; La Morgia and Venturino 2017).

For mortalities, the average lifetimes are taken from (Amori et al. 2008), and give for *Lepus europaeus* a range of 5–6 years, for *Sylvilagus floridanus* it is about 15 months, with individuals living up to 5 years, and for *Vulpes vulpes* it seldom exceeds 3–4 years. We therefore take the following averages

16 $$\begin{aligned} m=\frac{1}{3.5}= 0.28571, \quad n=\frac{1}{1.25}= 0.80000, \quad p=\frac{1}{5.5}= 0.18182. \end{aligned}$$

For the conversion coefficient *e*, i.e. the benefit that foxes obtain hunting their prey, as mentioned in Introduction, we set $e=0.91$.

To assess the reproduction rate $r_P$ for a generic population *P*, we consider the following equation:

$$\begin{aligned} {\dot{P}}=r_P P \end{aligned}$$

whose solution is:

$$\begin{aligned} P(t)=e^{r_Pt} P(0). \end{aligned}$$

Now, each female fox gives to life an average of 4 offsprings so that the foxes population will triplicate in 1 year: $P(1)=3P(0)$. The foxes population growth is thus obtained solving $3P(0)=e^{r}P(0)$ for *r* and obtaining $r=\log 3$. Data on the densities of fox populations in Italy are scanty. They probably range between 1.0-2.5 foxes per km$^2$ (Boitani and Prigioni 2003). Here we use a conservative value of 1.0 fox per km$^2$. Using (16) and solving for the intraspecific competition term from the equilibrium $E_1$, we find

$$\begin{aligned} c_{VV}=\log 3 - \frac{1}{3.5}= 0.81290. \end{aligned}$$

For the cottontails, the biologist data indicate that the cottontails population in Northern Italy after the reproductive period increases 4.5 times; this of course already discounts mortality, so that we can take $P(1)=4.5 P(0)$, giving $s=\log 4.5$. The maximum density recorded in lowland in Piedmont, the same region where the study of Cerri et al. (2017) was performed, is 110 cottontails per km$^2$ (Bertolino et al. 2011a). Since this value was only from an area, we use instead 100 cottontails per km$^2$. We then find the intraspecific competition coefficient from the equilibrium $E_2$ and (16),

$$\begin{aligned} c_{SS}=\frac{1}{100} \left[ \log 4.5 - \frac{1}{1.25} \right] = 0.0070408. \end{aligned}$$

The hares reproduction is highly variable; each female can give birth to 1–4 (with a mean of 2.6) juveniles and reproduces 2–5 times a year. We considered a mean of 2.6 juveniles with 3 reproductions and an annual output of 8 young ones. Hence, the population quintuplicates in 1 year, $P (1) = 5P (0)$; we thus set the reproduction as $u = \log 5$. The carrying capacity for the European hare is 26–40 hares per km$^2$ in territories with a good suitability (Pandini et al. 1998), i.e. mixed crops with wheat, meadows and other cultures. Taking 30 hares per km$^2$ and combining $E_4$ with (16), we thus find the intraspecific competition rate

$$\begin{aligned} c_{LL}=\frac{1}{30} \left[ \log 5 - \frac{1}{5.5} \right] = 0.047587. \end{aligned}$$

**Table 3:** The fixed parameter values used in all the simulations

Species	Parameters	Values
	*r*	$\log 3$
Foxes	*m*	$\frac{2}{7}$
	$c_{VV}$	$\log (3) - \frac{2}{7}$
	*e*	0.91
	*s*	$\log 4.5$
Cottontails	*n*	$\frac{4}{5}$
	$c_{SS}$	$\frac{\log (4,5)}{100} - \frac{4}{500}$
	*u*	$\log 5$
Hares	*p*	$\frac{2}{11}$
	$c_{LL}$	$\frac{\log (5)}{30} - \frac{2}{330}$

### The Bifurcations Chains

We provide now a few figures that relate some of the transcritical bifurcations to each other, where the transitions between one equilibrium and the next one depend on one specific model parameter. The parameter reference values are those of Table 3. For each case instead we provide the remaining parameters.

**The transition** $$E_7-E_3-E_7-E_5$$

Taking as bifurcation parameter *a*, the hunting rate on cottontails, for its increasing values we find three bifurcations, located at

$$\begin{aligned} a_{3,7}^1 = 1.0078, \quad a_{3,7}^2 = 2.0762, \quad a_{5,7} = 4.8392. \end{aligned}$$

The remaining parameter values are here

17 $$\begin{aligned} b=3.3, \quad V(0)=0.1, \quad S(0)=10, \quad L(0)=3. \end{aligned}$$

**Fig. 2:**
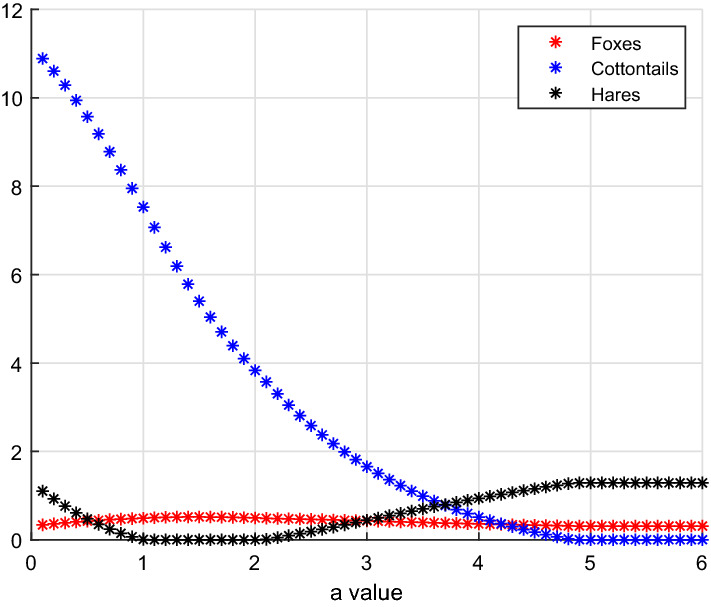
(Color Figure Online) For small values of *a*, we find coexistence, equilibrium $E_7$, then at $a_{3,7}^1=1.0078$ the hares vanish giving equilibrium $E_3$, subsequently in the interval $[a_{3,7}^2=2.0762, a_{5,7}=4.8392]$ hares reappear, and coexistence is re-established. Finally, for values of *a* exceeding $a_{5,7}=4.8392$, cottontails are wiped out and the system attains equilibrium $E_5$ (Color Figure Online)

In turn, the three-species interaction moves from coexistence, to the point where hares vanish, to coexistence again, and finally to the foxes–hares subsystem, see Fig. 2.

**The transition** $$E_5-E_1$$

**Fig. 3:**
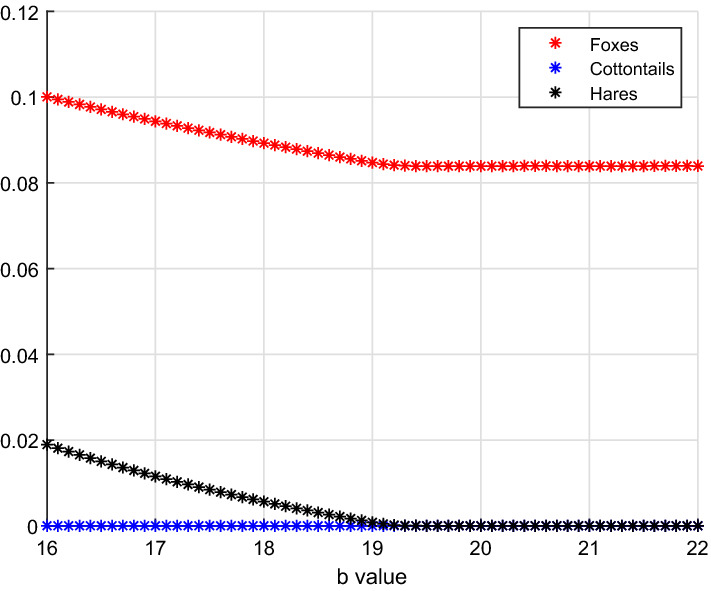
(Color Figure Online) Initially for $b\approx 16$, we find equilibrium $E_5$ where both foxes and hares coexist. Past the critical value $b_{1,5}=19.1891$, hares are wiped out, and only foxes survive at equilibrium $E_1$ (Color Figure Online)

Here the bifurcation parameter is *b*, the hunting rate on hares, in the range [16, 22]. For its increasing values, the three-species system moves from coexistence of both foxes and hares to the situation in which only foxes thrive, in view of the possibility of using other food supplies, Fig. 3. The specific parameter values are:

18 $$\begin{aligned} a=29, \quad V(0)=0.1, \quad S(0)=10, \quad L(0)=3. \end{aligned}$$

**The transition** $$E_3-E_1$$

**Fig. 4:**
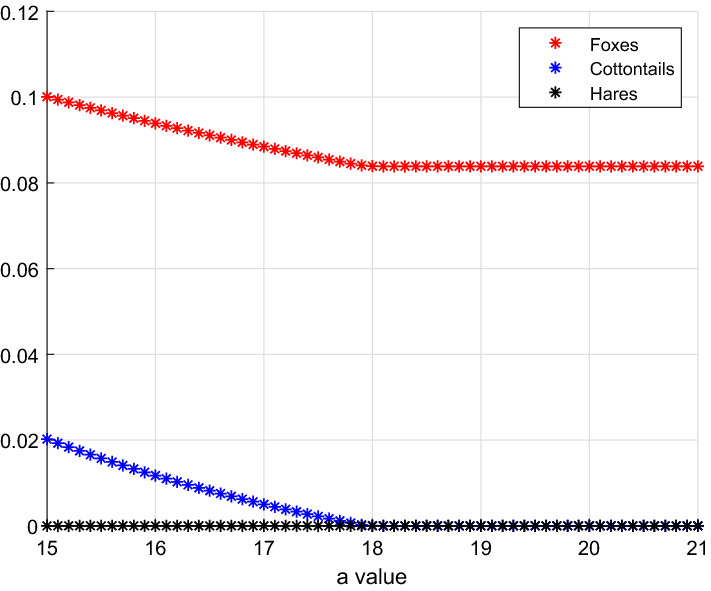
(Color Figure Online) Low values of *a* give equilibrium $E_3$ where both foxes and cottontails coexist. At the threshold $a_{1,3}=17.9329$, cottontails vanish and only foxes remain, at equilibrium $E_1$ (Color Figure Online)

Taking again *a* as an increasing bifurcation parameter, the three-species system moves from foxes–cottontails coexistence, to the point at which only foxes thrive, Fig. 4. Parameter values used:

19 $$\begin{aligned} b=40, \quad V(0)=0.1, \quad S(0)=10, \quad L(0)=3. \end{aligned}$$

**The transition** $$E_7-E_5-E_7-E_3$$

**Fig. 5:**
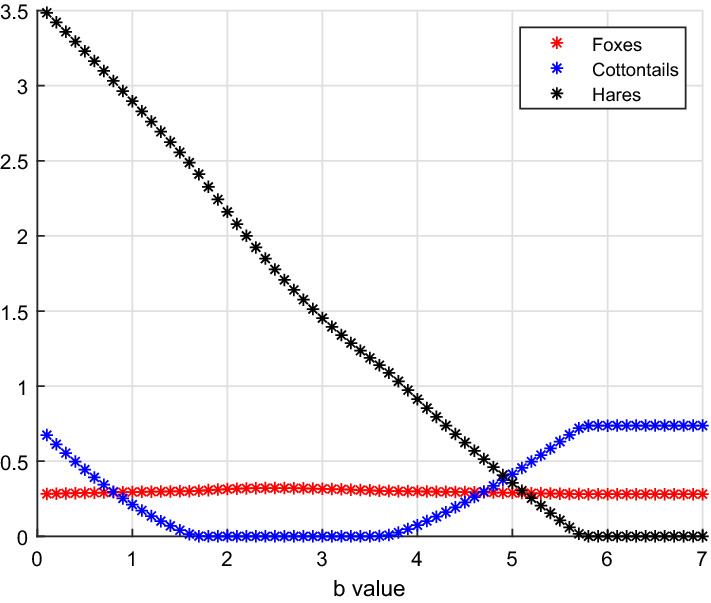
(Color Figure Online) For *b* small, coexistence is obtained, equilibrium $E_7$. At $b_{5,7}^1=1.6581$ cottontails disappear, leaving hares and foxes in the environment, then at $b_{5,7}^2=3.6921$ they reappear, so that coexistence of the three species is re-established, finally at $b_{7,3}=5.7296$ the hares are wiped out, and cottontails thrive together with foxes (Color Figure Online)

Here we take once more the hunting rate on hares *b* as varying parameter, Fig. 5. There are three critical values,

$$\begin{aligned} b_{5,7}^1 = 1.6581, \quad b_{5,7}^2 = 3.6921, \quad b_{3,7} = 5.7296 \end{aligned}$$

for which the three-species system moves from coexistence to the hares–foxes subsystem, to coexistence again, and finally to the cottontails–foxes subsystem. The remaining parameters here are:

20 $$\begin{aligned} a=5, \quad V(0)=0.1, \quad S(0)=10, \quad L(0)=3. \end{aligned}$$

**The transition** $$E_5-E_7-E_3$$

**Fig. 6:**
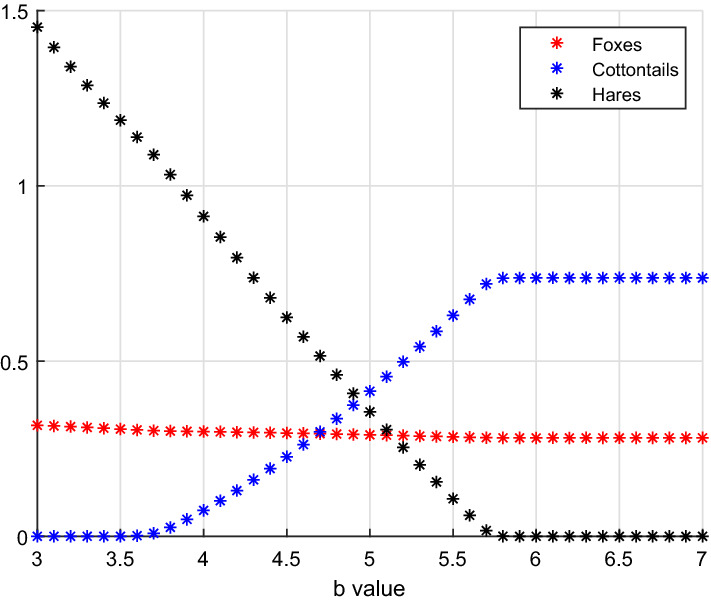
(Color Figure Online) Small values of *b* deprive the three-species system from the cottontails, equilibrium $E_5$. At about $b_{5,7} = 3.6921$, cottontails invade the system and coexistence is established, equilibrium $E_7$. For higher values of the hunting rate on hares, namely $b_{3,7} = 5.7296$ approximately, hares are wiped out (Color Figure Online)

The bifurcation parameter *b*, the predation rate on hares, in the interval [3, 7] generates the bifurcations for which the system undergoes a transition from $E_5$, the cottontails-free equilibrium, to $E_7$, coexistence, and finally to $E_3$, the hares-free point, Fig. 6. The other parameters are:

21 $$\begin{aligned} a=5, \quad V(0)=0.1, \quad S(0)=10, \quad L(0)=3. \end{aligned}$$

Note that this trend is biologically interesting, because it reflects a possible behaviour observed in the field studies. Indeed, in the areas under observation, the cottontails are an invasive species that modified the natural predator–prey dynamics. The results of the field study (Cerri et al. 2017) indicate that a larger cottontails population drives hares to extinction. As it can be seen from Fig. 6, increasing the predation rate on hares, the cottontails population increases and finally replaces the hares, because the latter are subject to an increasing hunting rate by the foxes, and are therefore driven to extinction.

## Discussion

The question raised in Introduction has therefore an answer.

**Fig. 7:**
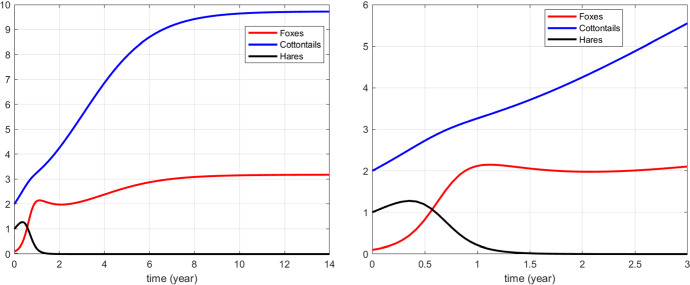
(Color Figure Online) As the cottontails invade, the hares are progressively wiped out by the foxes. Left: the long-range behaviour; Right: zoom for the initial stages of the invasion (Color Figure Online)

Note that with the parameter values

22 $$\begin{aligned} a=0.2, \quad b=3.0, \quad V(0)=0.1, \quad S(0)=2, \quad L(0)=1 \end{aligned}$$

the invasion mechanism becomes apparent. The cottontail population increases steadily, and following it, also the foxes attain higher population values. In turn, their damage to the hares increases, with the latter dropping and finally becoming extinct, see Fig. 7. In particular in the figure one can observe that while the cottontail population invades, growing from low values at the early times to a larger population around time $t=0.4$, the foxes population also raises up. Thus, their pressure on their prey increases, but the hares suffer more, their numbers decrease and eventually they are wiped out. Note that this happens even though the predation rate on cottontails exceeds the one on hares, $b=3.5 > a= 1.5$.

To further and strengthen our analysis, we try to relate our findings to the ones coming from field observations in Cerri et al. (2017). In particular, we found a relationship between hares and foxes similar to the one described in the last three figures of Cerri et al. (2017), by running a set of numerical simulations with randomly generated parameter values, so as to mimic the outcome of the field data gathered over an 8 year timespan.

We thus ran 500 simulations of the model (1), taking uniform random values for all the parameters, located in intervals of semiwidth 9% above and below the reference values of Table 3 supplemented by the values indicated here:

23 $$\begin{aligned} a=0.2, \quad b=0.5, \quad V(0)=0.4, \quad S(0)=1, \quad L(0)=3 . \end{aligned}$$

**Fig. 8:**
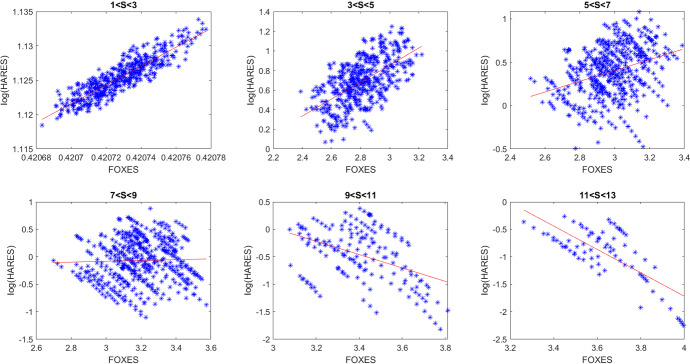
(Color Figure Online) Logarithm of hares population versus foxes. Left to right and top to bottom, each frame is related to higher values of cottontails densities. Specifically, top: $1<S<3$, $3<S<5$, $5<S<7$; bottom: $7<S<9$, $9<S<11$, $11<S<13$ (Color Figure Online)

**Fig. 9:**
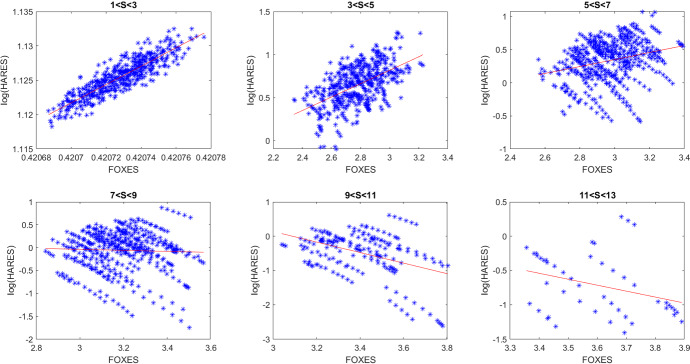
(Color Figure Online) Logarithm of hares population versus foxes. Left to right and top to bottom, each frame is related to higher values of cottontails densities. Specifically, top: $1<S<3$, $3<S<5$, $5<S<7$; bottom: $7<S<9$, $9<S<11$, $11<S<13$ (Color Figure Online)

The steady states were then evaluated and recorded for each simulation, together with the respective parameter values that generated them. Finally, regression lines were evaluated, and the results plotted again for the logarithm of the hares population versus the foxes. Two selected such results are reported in Figs. 8, 9, corresponding to two different random parameter choices. The various frames, left to right, report the findings for increasing values of the cottontails density. The plots represent the logarithm of the hares populations versus the foxes. There is a remarkable resemblance with the analogous plot obtained by the field data and reported in Cerri et al. (2017).

The transition $E_7 - E_3 - E_7 - E_5$ shows a situation where the hunting rate of foxes on cottontails is not limited and continues to increase over time. In this case, the system moves from an initial situation where predation of foxes on the introduced species is limited and the three species coexist to a final outcome where cottontails are wiped out and only the two native species remain. Such a situation would be positive for biodiversity because a native predator would thrive to extinction the introduced lagomorph. However, the red fox is a generalist predator, (Soe et al. 2017), and although the cottontails became an important prey of foxes in Italy (Balestrieri et al. 2006), the availability of many other food resources (e.g. rodents, insects, fruits) probably prevents the predator from increasing the predation rate beyond a certain limit.

Conversely with the transition $E_7 - E_5 - E_7 - E_3$, the hare declines rapidly starting from a very high and unrealistic density. The cottontail goes extinct and then recovers when the hare is at low density. This would indicate a strong predation pressure on the hare and a competitive effect of hares on cottontails, which can recolonize the area only when the hare is at low density. It is known that the fox cannot limit hares so drastically and that hares do not exclude cottontails (Bertolino et al. 2011a).

The transitions $E_5 - E_1$ and $E_3 - E_1$ indicate that the fox predation pressure on cottontails and hares is so high as to bring the two prey species to extinction. This situation is not supported by earlier field research. In areas where only the two native species are present, the hare is one of the food sources for foxes. However, predation is not considered to be a limiting factor for the lagomorph (Boitani and Prigioni 2003; Cerri et al. 2017). Indeed, in places where the hare has disappeared and the cottontail is the only widespread lagomorph, the American species becomes a main prey of foxes, but populations survive due to their high reproduction rates (Balestrieri et al. 2006).

The transition $E_5 - E_7 - E_3$ better reflects the results of previous research (Cerri et al. 2017). The system undergoes a transition from an alien species-free system to a one invaded by the introduced cottontail; subsequently, it evolves to a coexistence regime between the three species and finally the hare becomes extinct. For the parameter values (22), the simulations of this model show an agreement with the invasion process recorded on the field (Cerri et al. 2017). The simulation in Fig. 7 describes the invasion of an area by cottontail with a rapid increase in its population and a benefit for foxes that exploit this new prey and in turn increase their density. The predation pressure on both the hares and the cottontails consequently increases, but the hares suffer more and eventually go extinct. The cottontail better supports the higher foxes predation rate, due to its higher reproductive output (Chapman et al. 1980).

## Conclusion

In this work we were able to simulate a three-species system composed of two prey species, one native and one introduced, and their relations with a native predator. Our results mathematically describe the negative effect on the native European hare after the introduction of the invasive Eastern cottontail, mediated by an increased predation rate by foxes (Cerri et al. 2017). In Cerri et al. (2017) two nonexclusive hypotheses were discussed to explain the pattern observed when cottontail populations increase. In the first hypothesis, an increase in cottontail abundance would lead to a numerical response of foxes, magnifying their predatory impact on hares (a sort of “hyperpredation”, a particular case of apparent competition). The second hypothesis assumes that cottontails attract foxes in patches of permanent cover where they live. Since these habitats are also important resting sites for adult and young hares (Boitani and Prigioni 2003), an increased presence of foxes is likely to result in a higher predation risk for hares. Our simulation describes an increase in foxes’ densities after the invasion of cottontail, supporting the first hypothesis from Cerri et al. (2017) based on a hyperpredation of increasing fox populations on native hares.

## Appendix

**Boundedness details**

For an arbitrary $\eta >0$, using the equations in (1), we have

$$\begin{aligned} \frac{{\text {d}}A}{{\text {d}}t}+\eta A =&V \left( r-m+\eta - c_{VV} V \right) +S \left( s-n+\eta - c_{SS} S \right) +aSV(e-1)\\&+L \left( u-p+\eta - c_{LL} L \right) +bLV(e-1). \end{aligned}$$

Taking $e<1$ and evaluating the maxima $V_m$, $S_m$, $L_m$ of the three parabolae on the right, we, respectively, obtain

$$\begin{aligned} V_m:=\frac{1}{4 c_{VV}}(r-m+\eta )^2, \quad S_m:=\frac{1}{4 c_{SS}}(s-n+\eta )^2, \quad L_m:=\frac{1}{4 c_{LL}}(u-p+\eta )^2. \end{aligned}$$

Thus, the upper bound is found for which

24 $$\begin{aligned} \frac{{\text {d}}A}{{\text {d}}t}+\eta A \le V_m+S_m+L_m= C. \end{aligned}$$

Upon integration of this differential inequality, the final desired bound is obtained:

$$\begin{aligned} A(t)\le \max \left\{ \frac{C}{\eta },A(0) \right\} . \end{aligned}$$

**Global stability details**

**Equilibrium** $E_0$ In this case we set

$$\begin{aligned} {\mathcal {L}}_0=cV + gS+fL \end{aligned}$$

where *c*, *g* and *f* are arbitrary positive coefficients. Differentiating ${{{\mathcal {L}}}}_0$ with respect to time, we obtain, recalling (15),

$$\begin{aligned} \frac{{\text {d}} {\mathcal {L}}_0}{{\text {d}}t}= & {} c \frac{{\text {d}}V}{{\text {d}}t} + g \frac{{\text {d}}S}{{\text {d}}t} + f \frac{{\text {d}}L}{{\text {d}}t} =P^TAP+cV(r-m)+gS(s-n)+fL(u-p)\\\le & {} P^TAP \end{aligned}$$

where the last inequality follows on using the stability conditions (2). Now, choosing $f=g=e$ and $c=1$, *A* becomes diagonal, with negative eigenvalues; hence, it is negative definite as required.

**Equilibrium** $E_1$ Here we set

$$\begin{aligned} {\mathcal {L}}_1=cV_1 \left( \frac{V-V_1}{V_1}-\ln \left( 1+\frac{V-V_1}{V_1} \right) \right) +gS+fL . \end{aligned}$$

Upon differentiation, we find

$$\begin{aligned} \frac{{\text {d}} {\mathcal {L}}_1}{{\text {d}}t}= & {} c \frac{{\text {d}}V}{{\text {d}}t} -c \frac{V_1}{V} \frac{{\text {d}}V}{{\text {d}}t} + g \frac{{\text {d}}S}{{\text {d}}t} + f \frac{{\text {d}}L}{{\text {d}}t}\\= & {} P^TAP+gS(s-n-aV_1)+fL(u-p-bV_1) \le P^TAP, \end{aligned}$$

having used the stability conditions (4) in the last step. Choosing $f=g=e$ and $c=1$, *A* is seen to have negative eigenvalues, and in turn is negative definite. Global stability of $E_1$ therefore follows.

**Equilibrium** $E_2$ The candidate function is

$$\begin{aligned} {\mathcal {L}}_2=cV+gS_2 \left( \frac{S-S_2}{S_2}-\ln \left( 1+\frac{S-S_2}{S_2} \right) \right) +fL \end{aligned}$$

and its differentiation gives

$$\begin{aligned} \frac{{\text {d}} {\mathcal {L}}_2}{{\text {d}}t}= & {} c \frac{{\text {d}}V}{{\text {d}}t} + g \frac{{\text {d}}S}{{\text {d}}t} - g \frac{S_2}{S} \frac{{\text {d}}S}{{\text {d}}t} + f \frac{{\text {d}}L}{{\text {d}}t}\\ \quad= & {} P^TAP+cV(r-m+eaS_2)+fL(u-p) \le P^TAP, \end{aligned}$$

which follows using (5). Negative definiteness is then obtained by the choice $f=g=e$ and $c=1$.

**Equilibrium** $E_3$ We now set

$$\begin{aligned} {\mathcal {L}}_3= & {} cV_3 \left( \frac{V-V_3}{V_3}-\ln \left( 1+\frac{V-V_3}{V_3} \right) \right) \\+ & {} gS_3 \left( \frac{S-S_3}{S_3} - \ln \left( 1+\frac{S-S_3}{S_3} \right) \right) +fL \end{aligned}$$

Differentiating with the help of (8), we obtain

$$\begin{aligned} \frac{{\text {d}} {\mathcal {L}}_3}{{\text {d}}t}= & {} c \frac{{\text {d}}V}{{\text {d}}t} -c \frac{V_3}{V} \frac{{\text {d}}V}{{\text {d}}t} + g \frac{{\text {d}}S}{{\text {d}}t}\\- & {} g \frac{S_3}{S} \frac{{\text {d}}S}{{\text {d}}t} + f \frac{{\text {d}}L}{{\text {d}}t} =P^TAP+fL(u-p-bV_3) \le P^TAP . \end{aligned}$$

Choosing $f=g=e$ and $c=1$, *A* turns to be negative definite.

**Equilibrium** $E_4$ We now choose

$$\begin{aligned} {\mathcal {L}}_4=cV+gS+fL_4 \left( \frac{L-L_4}{L_4}- \ln \left( 1+\frac{L-L_4}{L_4} \right) \right) \end{aligned}$$

which, together with (6), gives

$$\begin{aligned} \frac{{\text {d}} {\mathcal {L}}_4}{{\text {d}}t}= & {} c \frac{{\text {d}}V}{{\text {d}}t} + g \frac{{\text {d}}S}{{\text {d}}t} + f \frac{{\text {d}}L}{{\text {d}}t} \\- & {} f \frac{L_4}{L} \frac{{\text {d}}L}{{\text {d}}t}=P^TAP+cV(r-m+ebL_4)+gS(s-n) \le P^TAP. \end{aligned}$$

Global stability follows by setting $f=g=e$ and $c=1$.

**Equilibrium** $E_5$ The candidate Lyapunov function here is set as follows:

$$\begin{aligned} {\mathcal {L}}_5= & {} cV_5 \left( \frac{V-V_5}{V_5}-\ln \left( 1+\frac{V-V_5}{V_5}\right) \right) \\+ & {} gS +fL_5 \left( \frac{L-L_5}{L_5}-\ln \left( 1+\frac{L-L_5}{L_5} \right) \right) . \end{aligned}$$

Differentiating and using (10), we obtain

$$\begin{aligned} \frac{{\text {d}} {\mathcal {L}}_5}{{\text {d}}t}= & {} c \frac{{\text {d}}V}{{\text {d}}t} -c \frac{V_5}{V} \frac{{\text {d}}V}{{\text {d}}t} + g \frac{{\text {d}}S}{{\text {d}}t}\\+ & {} f \frac{{\text {d}}L}{{\text {d}}t} - f \frac{L_5}{L} \frac{{\text {d}}L}{{\text {d}}t} =P^TAP+gS(s-n-aV_5) \le P^TAP . \end{aligned}$$

Global stability now follows by setting $f=g=e$ and $c=1$.

**Equilibrium** $E_6$ In this case the candidate is

$$\begin{aligned} {\mathcal {L}}_6= & {} cV+gS_6 \left( \frac{S-S_6}{S_6}-\ln \left( 1+\frac{S-S_6}{S_6}\right) \right) \\+ & {} fL_6\left( \frac{L-L_6}{L_6}-\ln \left( 1+\frac{L-L_6}{L_6}\right) \right) \end{aligned}$$

so that, using (12),

$$\begin{aligned} \frac{{\text {d}} {\mathcal {L}}_6}{{\text {d}}t}= & {} c \frac{{\text {d}}V}{{\text {d}}t} + g \frac{{\text {d}}S}{{\text {d}}t} -g \frac{S_6}{S} \frac{{\text {d}}S}{{\text {d}}t}\\+ & {} f \frac{{\text {d}}L}{{\text {d}}t} - f \frac{L_6}{L} \frac{{\text {d}}L}{{\text {d}}t} =P^TA P+cV(r-m+eaS_6+ebL_6) \le P^TA P . \end{aligned}$$

We finally choose $f=g=e$ and $c=1$ to obtain negative definiteness.

**Equilibrium** $E_7$ The candidate function is here

$$\begin{aligned} {\mathcal {L}}_7= & {} cV_7 \left( \frac{V-V_7}{V_7}-\ln \left( 1+\frac{V-V_7}{V_7}\right) \right) +gS_7 \left( \frac{S-S_7}{S_7}-\ln \left( 1+\frac{S-S_7}{S_7}\right) \right) \\+ & {} fL_7 \left( \frac{L-L_7}{L_7}-\ln \left( 1+\frac{L-L_7}{L_7} \right) \right) \end{aligned}$$

giving

$$\begin{aligned} \frac{{\text {d}} {\mathcal {L}}_7}{{\text {d}}t}= & {} c \frac{{\text {d}}V}{{\text {d}}t} -c \frac{V_7}{V} \frac{{\text {d}}V}{{\text {d}}t} + g \frac{{\text {d}}S}{{\text {d}}t} \\- & {} g \frac{S_7}{S} \frac{{\text {d}}S}{{\text {d}}t} + fs \frac{{\text {d}}L}{{\text {d}}t} - f \frac{L_7}{L} \frac{{\text {d}}L}{{\text {d}}t} =P^TAP . \end{aligned}$$

The choice $f=g=e$ and $c=1$ renders *A* negative definite, and thus, global stability is achieved.

**Bifurcations**

**Bifurcation** $E_0-E_1$ At $E_0$, imposing the critical value $r_{0,1}=m$, one eigenvalue vanishes and $E_0=E_1$. Now $v=(1,0,0)^T$ is the eigenvector of $J(E_0,r_{0,1})$,

$$\begin{aligned} J(E_0,r_{0,1})= \begin{bmatrix} 0 &{} 0 &{} 0\\ 0 &{} s-n &{} 0\\ 0 &{} 0 &{} u-p \end{bmatrix} \end{aligned}$$

while $w=(1,0,0)^T$ is the one of $[J(E_0,r_{0,1})]^T$. A transcritical bifurcation then exists between $E_0$ and $E_1$ for $r_{0,1}=m$ in view of:

$$\begin{aligned} F_r(E_0,r_{0,1})= & {} (0,0,0)^T, \quad w^T F_r(E_0,r_{0,1}) = 0, \\ w^T(DF_r v)= & {} 1\ne 0, \quad w^T(D^2F(E_0,r_{0,1})(v,v))=- 2 c_{VV}\ne 0. \end{aligned}$$

**Bifurcation** $E_0-E_2$ Here we impose the threshold value $s_{0,2}=n$, to get a vanishing eigenvalue and $E_0=E_2$. The eigenvectors of $J(E_0,s_{0,2})$ and its transpose are, respectively, $v=(0,1,0)^T$ and $w=(0,1,0)^T$. A transcritical bifurcation occurs because of

$$\begin{aligned} F_s(E_0,s_{0,2})= & {} (0,0,0)^T, \quad w^T F_s(E_0,s_{0,2}) = 0, \\ w^T(DF_s v)= & {} 1\ne 0, \quad w^T(D^2F(E_0,s_{0,2})(v,v))=- 2 c_{SS}\ne 0 . \end{aligned}$$

**Bifurcation** $E_0-E_3$ In this case we set $u_{0,4}=p$ and find $E_0=E_4$. The required eigenvalues of $J(E_0,u=p)$ and $[J(E_0,u_{0,4})]^T$ are $v=(0,0,1)^T$ and $w=(0,0,1)^T$. The transcritical bifurcation arises in view of

$$\begin{aligned} F_u(E_0,u_{0,4})= & {} (0,0,0)^T, \quad w^T F_u(E_0,u_{0,4}) = 0, \\ w^T(DF_u v)= & {} 1\ne 0, \quad w^T(D^2F(E_0,u_{0,4})(v,v))=- 2 c_{LL}\ne 0 . \end{aligned}$$

**Bifurcation** $$ E_1-E_3$$ To verify the transcritical bifurcation in this case to have $$E_1=E_3$$, we set

$$\begin{aligned} a_{1,3}=\frac{s-n}{V_1}= c_{VV} \frac{s-n}{r-m}. \end{aligned}$$

The required eigenvectors are then $v=(e(s-n),r-m,0)^T$ and $w=(0,1,0)^T$, and the following conditions hold:

$$\begin{aligned}&F_a(E_1,a_{1,3})=(0,0,0)^T, \quad w^T F_a(E_1,a_{1,3}) = 0, \quad w^T(DF_a v)=-V_1(r-m)\ne 0, \\&w^T(D^2F(E_1,a_{1,3})(v,v))= (r-m) \left. [-2 c_{SS} (r-m) - 2ae (s-n)]\right| _{E_1,a_{1,3}=0} \\&\quad \, =-2 c_{SS} (r-m)^2\ne 0, \\&w^T(D^2F(E_1,a_{1,3})(v,v))=-ae (s-n) (r-m) -2 c_{SS} (r-m)^2 \ne 0. \end{aligned}$$

**Bifurcation** $E_1-E_5$ To get $E_1=E_5$, let

$$\begin{aligned} b_{1,5}=\frac{u-p}{V_1}=c_{VV}\frac{u-p}{r-m}. \end{aligned}$$

The eigenvectors of the Jacobian and its transpose at this point with the parameter choice $b=b_{1,5}$ are, respectively, $v=(e(u-p),0,r-m)^T$ and $w=(0,0,1)^T$. The transcritical bifurcation is obtained in view of the conditions

$$\begin{aligned}&F_b(E_1,b_{1,5})=(0,0,0)^T, \quad w^T F_b(E_1,b_{1,5}) = 0, \quad w^T(DF_b v)=-V_1(r-m)\ne 0, \\&w^T(D^2F(E_1,b_{1,5})(v,v))=-2(r-m) \left. [be (u-p)+c_{LL} (r-m)] \right| _{E_1,b_{1,5}=0} \\&\quad \, = -2 c_{LL} (r-m)^2 \ne 0 . \end{aligned}$$

**Bifurcation** $E_2-E_3$ Here to obtain $E_2=E_3$ we choose $a_{2,3}=(m-r)(eS_2)^{-1}$ and find the eigenvectors $v=(e(n-s),r-m,0)^T$ and $w=(1,0,0)^T$, as well as the conditions

$$\begin{aligned}&F_a(E_2,a_{2,3})=(0,0,0)^T, \quad w^T F_a(E_2,a_{2,3}) = 0, \quad w^T(DF_a v)=e^2S_2(n-s)\ne 0, \\&w^T(D^2F(E_2,a_{2,3})(v,v))=\left. [-2 c_{VV} v_1^2+2ae v_1v_2]\right| _{E_2,a_{2,3}=0}\\&\quad =\left. [-2 c_{VV} e^2(n-s)^2+2ae (n-s)(r-m)] \right| _{E_2,a_{2,3}=0} = -2 c_{VV} e^2(n-s)^2 \ne 0 \end{aligned}$$

This indicates once more that a transcritical bifurcation indeed arises between these points.

**Bifurcation** $E_2-E_6$ Considering $u_{2,6}=u_{0,4}=p$, we have $E_2=E_6$ and the eigenvectors $v=(0,0,1)^T$ and $w=(0,0,1)^T$, and the conditions

$$\begin{aligned} F_u(E_2,u_{2,6})= & {} (0,0,0)^T, \quad w^T F_u(E_2,u_{2,6}) = 0, \\ w^T(DF_u v)= & {} 1\ne 0, \quad w^T(D^2F(E_2,u_{2,6})(v,v))=-2c_{LL}\ne 0 , \end{aligned}$$

thus showing the occurrence of the transcritical bifurcation.

**Bifurcation** $E_3-E_7$ To have $E_3=E_7$, set $b_{3,7} = (u-p)V_3^{-1}$ and obtain the following eigenvectors: $v=(2c_{SS}eb, -abe, 4 c_{VV}c_{SS}+a^2e )^T$ and $w=(0,0,1)^T$. The conditions for a transcritical bifurcation are then satisfied:

$$\begin{aligned} F_b(E_3,b_{3,7})= & {} (0,0,0)^T, \quad w^T F_b(E_3,b_{3,7}) = 0, \\ w^T(DF_b v)= & {} -V_3 v_3 = - V_3 [4c_{VV}c_{SS}+a^2e] \ne 0, \\ w^T(D^2F(E_3,b_{3,7})(v,v))= & {} -2 b_{3,7} v_1 v_3 - 2 c_{LL} v_3^2 = - 2 c_{LL} v_3 ^2 \ne 0 . \end{aligned}$$

**Bifurcation** $E_4-E_5$ Choosing $b_{4,5} =(m-r)(eL_4)^{-1}$ we obtain $E_4=E_5$ and the eigenvectors $v=(c_{LL},0,-b)^T$ and $w=(1,0,0)^T$, as well as the conditions for the transcritical bifurcation:

$$\begin{aligned}&F_b(E_4,b_{4,5})=(0,0,0)^T, \quad w^T F_b(E_4,b_{4,5}) = 0, \quad w^T(DF_b v)=e (u-p) \ne 0, \\&w^T(D^2F(E_4,b_{4,5})(v,v))= v_1 [-2 c_{VV} v_1 + 2eb_{4,5} v_3 ] = -2 c_{VV} c_{LL} ^2 \ne 0 . \end{aligned}$$

**Bifurcation** $E_4-E_6$ Here we take $s_{4,6}=n$ to have $E_4=E_6$, with the eigenvectors $v=(0,1,0)^T$ and $w=(0,1,0)^T$. The conditions that ensure the transcritical bifurcation are seen to hold:

$$\begin{aligned} F_s(E_4,s_{4,6})= & {} (0,0,0)^T, \quad w^T F_s(E_4,s_{4,6}) = 0, \\ w^T(DF_s v)= & {} 1\ne 0, \quad w^T(D^2F(E_4,s_{4,6})(v,v))=- 2 c_{SS} \ne 0 . \end{aligned}$$

**Bifurcation** $E_5-E_7$ The choice $a_{5,7} = (s-n)V_5^{-1}$ returns $E_5=E_7$ and the eigenvectors

$$\begin{aligned} v=( ae c_{LL} , c_{VV}c_{LL}+b^2e , -abe )^T, \quad w=(0,1,0)^T. \end{aligned}$$

The conditions for the transcritical bifurcation are satisfied:

$$\begin{aligned} F_a(E_5,a_{5,7})= & {} (0,0,0)^T, \quad w^T F_a(E_5,a_{5,7}) = 0, \\ w^T(DF_a v)= & {} -V_5 (c_{VV}c_{LL}+b^2e ) = be(u-p)+c_{LL}(r-m) \ne 0, \\ w^T(D^2F(E_5,a_{5,7})(v,v))= & {} -2a v_1 v_2 -2 c_{SS} v_2^2 = -2v_2 [a v_1 + c_{SS} v_2] \ne 0 . \end{aligned}$$

**Bifurcation** $E_6-E_7$ In this case taking

$$\begin{aligned} b_{6,7} = \frac{m-r}{eL_6}-\frac{aS_6}{L_6} \end{aligned}$$

which gives the eigenvectors

$$\begin{aligned} v=\left( c_{SS}c_{LL},-ac_{LL},-b c_{SS} \right) ^T, \quad w=(1,0,0)^T \end{aligned}$$

the conditions for the transcritical bifurcation are satisfied

$$\begin{aligned} F_b(E_6,b_{6,7})= & {} (0,0,0)^T, \quad w^T F_b(E_6,b_{6,7}) = 0, \\ w^T(DF_b v)= & {} eL_6 v_1 = e c_{SS} (u-p) \ne 0 , \\ w^T(D^2F(E_6,b_{6,7})(v,v))= & {} 2v_1 [ -c_{VV} v_1 + ae v_2 + eb_{6,7} v_3 ]\\= & {} -2 v_1 \left[ c_{VV} c_{SS}c_{LL} + a^2e c_{LL} \right] \quad \\= & {} -2 c_{SS}c_{LL} \left[ c_{VV} c_{SS}c_{LL} + a^2e c_{LL} \right] \ne 0 . \end{aligned}$$
